# Piloting Upfront Xpert MTB/RIF Testing on Various Specimens under Programmatic Conditions for Diagnosis of TB & DR-TB in Paediatric Population

**DOI:** 10.1371/journal.pone.0140375

**Published:** 2015-10-15

**Authors:** Neeraj Raizada, Kuldeep Singh Sachdeva, Soumya Swaminathan, Shubhangi Kulsange, Sunil D. Khaparde, Sreenivas Achuthan Nair, Ashwani Khanna, Kamal Kishore Chopra, Mahmud Hanif, Gulshan Rai Sethi, K. R. Umadevi, G. Keshav Chander, Brojakishore Saha, Amar Shah, Malik Parmar, Mayank Ghediya, Jyoti Jaju, Catharina Boehme, Chinnambedu Nainarappan Paramasivan

**Affiliations:** 1 Foundation for Innovative New Diagnostics, New Delhi, India; 2 Central TB Division, Government of India, New Delhi, India; 3 New Delhi TB Centre, New Delhi, India; 4 National Institute of research in Tuberculosis, Chennai, India; 5 Intermediate Reference Laboratory, Hyderabad, India; 6 Intermediate Reference Laboratory, Kolkata, India; 7 World Health Organization, Country Office for India, New Delhi, India; University College London, UNITED KINGDOM

## Abstract

**Background:**

India accounts for one-fifth of the global TB incidence. While the exact burden of childhood TB is not known, TB remains one of the leading causes of childhood mortality in India. Bacteriological confirmation of TB in children is challenging due to difficulty in obtaining quality specimens, in the absence of which diagnosis is largely based on clinical judgement. While testing multiple specimens can potentially contribute to higher proportion of laboratory confirmed paediatric TB cases, lack of high sensitivity tests adds to the diagnostic challenge. We describe here our experiences in piloting upfront Xpert MTB/RIF testing, for diagnosis of TB in paediatric population in respiratory and extra pulmonary specimens, as recently recommended by WHO.

**Method:**

Xpert MTB/RIF testing was offered to all paediatric (0–14 years) presumptive TB cases (both pulmonary and extra-pulmonary) seeking care at public and private health facilities in the project areas covering 4 cities of India.

**Results:**

Under this pilot project, 8,370 paediatric presumptive TB & presumptive DR-TB cases were tested between April and–November 2014. Overall, 9,149 specimens were tested, of which 4,445 (48.6%) were non-sputum specimens. Xpert MTB/RIF gave 9,083 (99.2%, CI 99.0–99.4) valid results. Of the 8,143 presumptive TB cases enrolled, 517 (6.3%, CI 5.8–6.9) were bacteriologically confirmed. TB detection rates were two fold higher with Xpert MTB/RIF as compared to smear microscopy. Further, a total of 60 rifampicin resistant TB cases were detected, of which 38 were detected among 512 presumptive TB cases while 22 were detected amongst 227 presumptive DR-TB cases tested under the project.

**Conclusion:**

Xpert MTB/RIF with advantages of quick turnaround testing-time, high proportion of interpretable results and feasibility of rapid rollout, substantially improved the diagnosis of bacteriologically confirmed TB in children, while simultaneously detecting rifampicin resistance.

## Background

India has the world’s largest burden of tuberculosis (TB) and accounts for one-fifth of the global TB incidence [1]. While, globally the exact burden of childhood TB is not well documented, it is estimated that childhood TB constitutes about 10–20% of all TB cases, in high burden countries [3–4] and TB remains one of the leading cause of childhood mortality [2]. In 2013, 63,919 paediatric TB cases were notified accounting for 5% of notified TB cases [5] in India, under the Revised National Tuberculosis Control Programme (RNTCP).

Diagnosis of pulmonary TB in children is challenging, more so in resource-limited, tuberculosis- endemic countries and is largely based on clinical and radiological findings and medical history [6–7]. Bacteriological confirmation of pulmonary TB is challenging due to difficulty in obtaining good quality sputum specimens from children. In the absence of quality specimens, one has to rely on testing alternate types of specimens from children. Here again confirmation of TB becomes challenging due to difficulties in obtaining these specimens, inadequate clinical sample volumes and paucibacillary nature of biological samples [8–9]. Diagnostic efforts are also undermined by the lack of diagnostic tests with high sensitivity that are simple to use and can be applied at the point of clinical care [8]. Isolation of *M tuberculosis* by culture, while considered as gold standard for diagnosing TB, takes 4–8 weeks and often requires expensive and sophisticated laboratory facilities which cannot be afforded in most resource-limited settings [10].

Though there are various PCR based diagnostic tests available for TB diagnosis, the specificities and sensitivities of these tests are known to be quite variable [8, 11]. Further, these tests involve multiple manual steps and long turnaround time, making them unsuitable for decentralised deployment. A series of meta-analyses have shown Xpert MTB/RIF (here after called as Xpert) assay (Cepheid Inc., Sunnyvale, California), a cartridge based nucleic acid amplification test (NAAT) to have a high specificity with variable sensitivity in different type of specimens for TB diagnosis [8, 12–14]. In 2013, World Health Organization (WHO) endorsed the use of Xpert assay for TB diagnosis in paediatric presumptive pulmonary and extra-pulmonary tuberculosis (EPTB) cases [15–16]. Xpert, a tool with a quick turn-around time, which simultaneously detects TB and rifampicin resistance, offers a promising solution to achieve the global objective of improved TB care and control and early TB case detection [17]. In line with 2013 WHO recommendations a pilot project was undertaken under RNTCP in four major cities of India, offering upfront Xpert testing to all types of paediatric presumptive TB and DR-TB cases. The primary objectives of the project were:

- To identify and document the feasibility of routine upfront implementation of Xpert assay for paediatric TB diagnosis.
- To evaluate Xpert assay performance in different types of paediatric specimens such as gastric lavage, BAL, induced sputum, lymph node aspirates, etc., under routine programmatic conditions.
- To assess the diagnostic yield of Xpert assay in terms of TB and DR-TB detection in different types of paediatric TB specimens (respiratory and extra-pulmonary) in comparison to smear microscopy under routine programme conditions.

## Methods

### Project Setting

The current pilot project provided upfront Xpert testing for presumptive paediatric TB and DR-TB cases by establishing high through put Xpert laboratories, within existing RNTCP reference laboratories. One such Xpert laboratories was established in each of the four cities, namely Chennai, Delhi, Hyderabad and Kolkata, covering a population of 14.3 million, for city wide project coverage. It may be noted here that while the exact paediatric population is not known, children < 15 years constitute about 25% of the population. Geographic coverage of the pilot was ensured by means of referral and free rapid specimen transport linkages between the Xpert lab and public and private institutions in the area. A number of sensitization workshops and advocacy meetings were organised with various health providers for participation under the current pilot. All the presumptive paediatric TB and DR-TB cases coming to collaborating facilities and hospitals (both public/private) were offered free of cost Xpert testing. Xpert test was performed on various types of specimen such as gastric lavage/aspirate, BAL, CSF, induced sputum, lymph node aspirates, etc.

### Project Design

**Fig 1:**
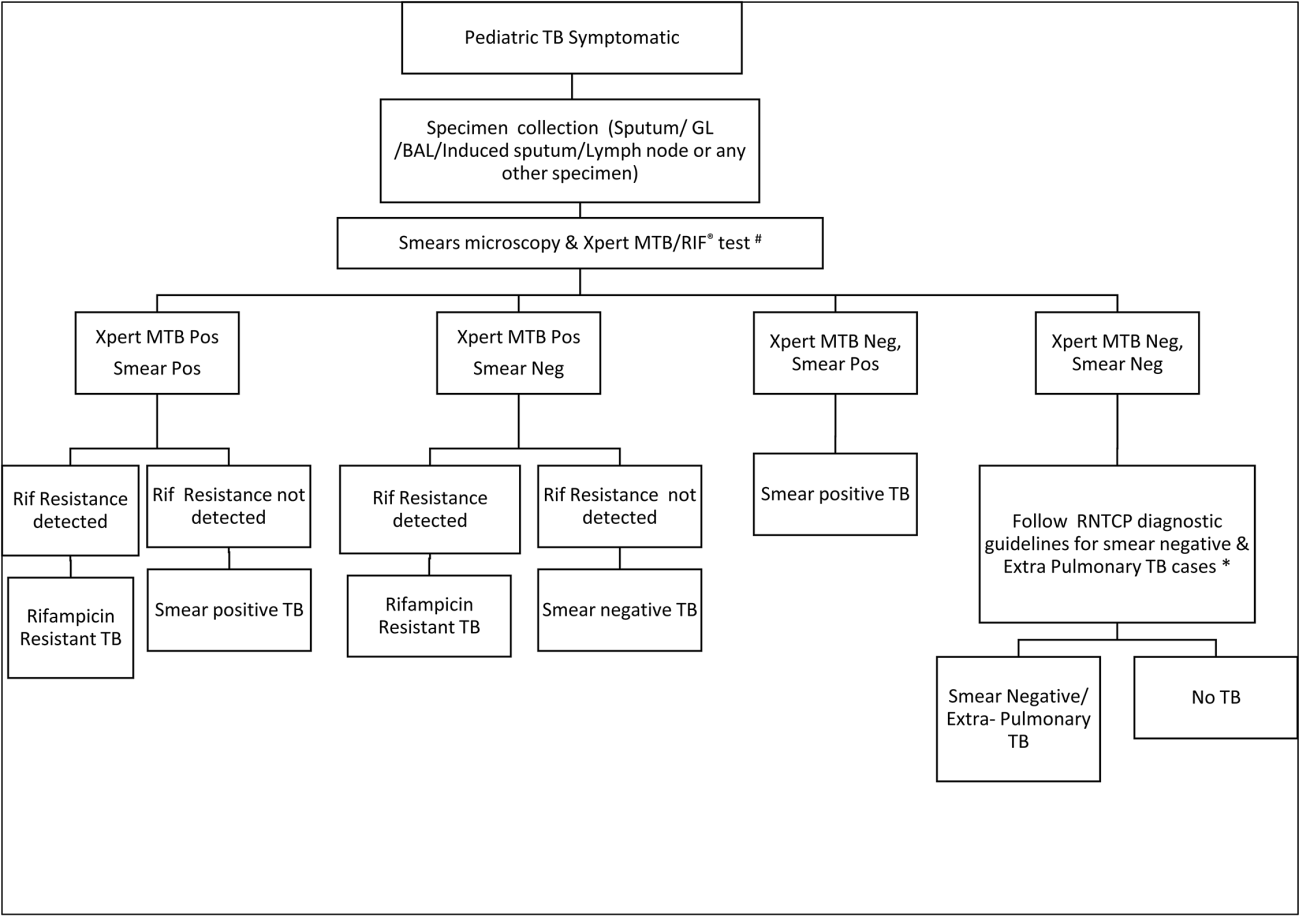
Project diagnostic algorithm.

Children (age 0–14 years) presenting with signs and symptoms suggestive of TB to any of the health facilities in the project area between April’14 to November’14 were prospectively enrolled in the pilot. Respiratory and extra-pulmonary specimens were collected at the respective health facilities and transported to the Xpert lab established in the city. The specimen transportation mechanisms were micro planned across all 4 cities, taking into account feasible local transportation mechanism acceptable to various health facilities. The mechanisms deployed for rapid specimen transportation included, using commercial courier services and local volunteers whose incidental costs were reimbursed at a standard rate. The average cost of transportation covered under the project was one USD per specimen shipment received at the lab. The referring providers provided their contact details in the test request form. Ziehl Neelsen (ZN) smear microscopy and Xpert testing was done for all patient specimens. A rapid reporting mechanism was established to ensure that all test results were promptly communicated to providers by e-mail and short messaging service (SMS). Standard diagnostic algorithm approved by RNTCP was followed under the pilot for patient management (Fig 1)

#### Definitions

Presumptive paediatric TB cases were defined as per the Indian RNTCP guidelines [16–17]. This includes children presenting with fever and/or cough for ≥2 weeks, with or without weight loss or no weight gain, or presenting with symptoms suggestive of pulmonary and/or extra-pulmonary TB.

Presumptive paediatric DR-TB cases were defined as previously-diagnosed paediatric TB cases based on smear-microscopy results and/or clinically; referred for drug susceptibility testing (DST) because of an elevated risk of drug-resistant TB. National guidelines used in the project define high-risk TB cases as those with previous history of anti-TB treatment, on treatment with positive sputum smear result at any follow up smear examination, diagnosed TB cases with HIV-co-infection, and contacts of a known MDR-TB case diagnosed with TB [18].

#### Specimen collection methods for different type of specimens

Standardised methodology for collection of various non-sputum specimens was followed. Specimens were collected at referral facilities which were linked with Xpert lab and equipped to perform different types of specimen collection procedures. The specimens were collected by trained medical personnel at these centres using standard methods.

#### Sample processing on Xpert MTB/RIF

Xpert testing was performed as per project diagnostic algorithm (Fig 1). In cases where specimens was less (<1ml), preference was given to Xpert testing ahead of smear microscopy in line with WHO recommendations [15–16]. For a given patient, whenever multiple types of specimen were available, all types of available specimen were tested. In case of ‘error’ and ‘no result’ test result on Xpert repeat test was performed on the remaining sample mix and in case of ‘invalid’ and ‘rifampicin resistance indeterminate’ test result, repeat testing was performed on a second specimen.

Sputum specimens were tested by adding of buffer in 1:2 proportions as recommended by the manufacturer **Invalid source specified.**. For non-sputum specimen, standard operating procedures (SOPs) developed by RNTCP [19] and WHO were adopted. Confirmatory DST for rifampicin resistant cases diagnosed on Xpert was performed on LPA and/or culture DST under the pilot. Confirmatory DST was performed either on the remnant specimen, or additional specimen, if available or on a freshly collected patient specimen. All the diagnosed TB and rifampicin resistant TB cases were initiated on appropriate anti TB regimens based on prior history of anti TB treatment as per RNTCP treatment guidelines [20].

#### Feasibility assessment

Feasibility of Xpert implementation was assessed both in terms of the ability of the assay to produce a valid result and the operational feasibility of providing upfront Xpert testing for paediatric presumptive TB cases though a single Xpert lab in each of the 4 cities.

The absence of a valid test result for any given assay initiated was defined as a ‘test failure’ regardless of the underlying reason. Information on frequency of various reasons for the occurrence of test failure and associated factors such as ambient temperature, power failure and or procedural error was collected and analysed.

The operational feasibility of offering upfront Xpert testing through referral and rapid specimen transport linkages between public and private institutions and the lab were assessed by analysing the turnaround time for specimen transportation, diagnosis and reporting of results to the provider.

### Data management

Data for all presumptive TB and DR-TB cases was collected from RNTCP lab request form (Annexure I). The pilot was carried out under uncontrolled programmatic field conditions covering health facilities in the selected geographic area. No additional patient related clinical data, including details of X-Ray, histo-pathological findings, etc. was collected. However, for rifampicin resistant TB cases diagnosed under the pilot, additional information regarding history of contact past history of TB and BCG vaccination status was retrospectively collected through personal visit and one to one interview. Data was analysed using Microsoft Excel 2013 and EpiData Analysis (Version 2.1). All confidence intervals were calculated based on the binomial distribution with a 95% probability interval. For analytical purpose, paediatric patients were categorized into 3 groups: 0–4 years, 5–9 years, and 10–14 years of age. Odd’s ratio was calculated to determine the statistical significance and relation between two variables.

### Ethical issues

The use of upfront Xpert testing for presumptive paediatric TB cases is an approved intervention under RNTCP. As such the results presented here are our experience sharing of the pilot of approved interventions in the programmatic settings within the existing accredited RNTCP TB diagnostic lab. Since the observations describe here are a part of implementation of approved interventions under RNTCP, a separate ethical clearance was not required.

## Results

The project was implemented between April to November 2014, starting with one city in April 2014 and scaled up to all four cities by May’14. Within 3 months of project roll out, an average of 1,300 presumptive paediatric TB and DR-TB cases were being tested per month across the four cities (Figure A in S1 File).

**Fig 2:**
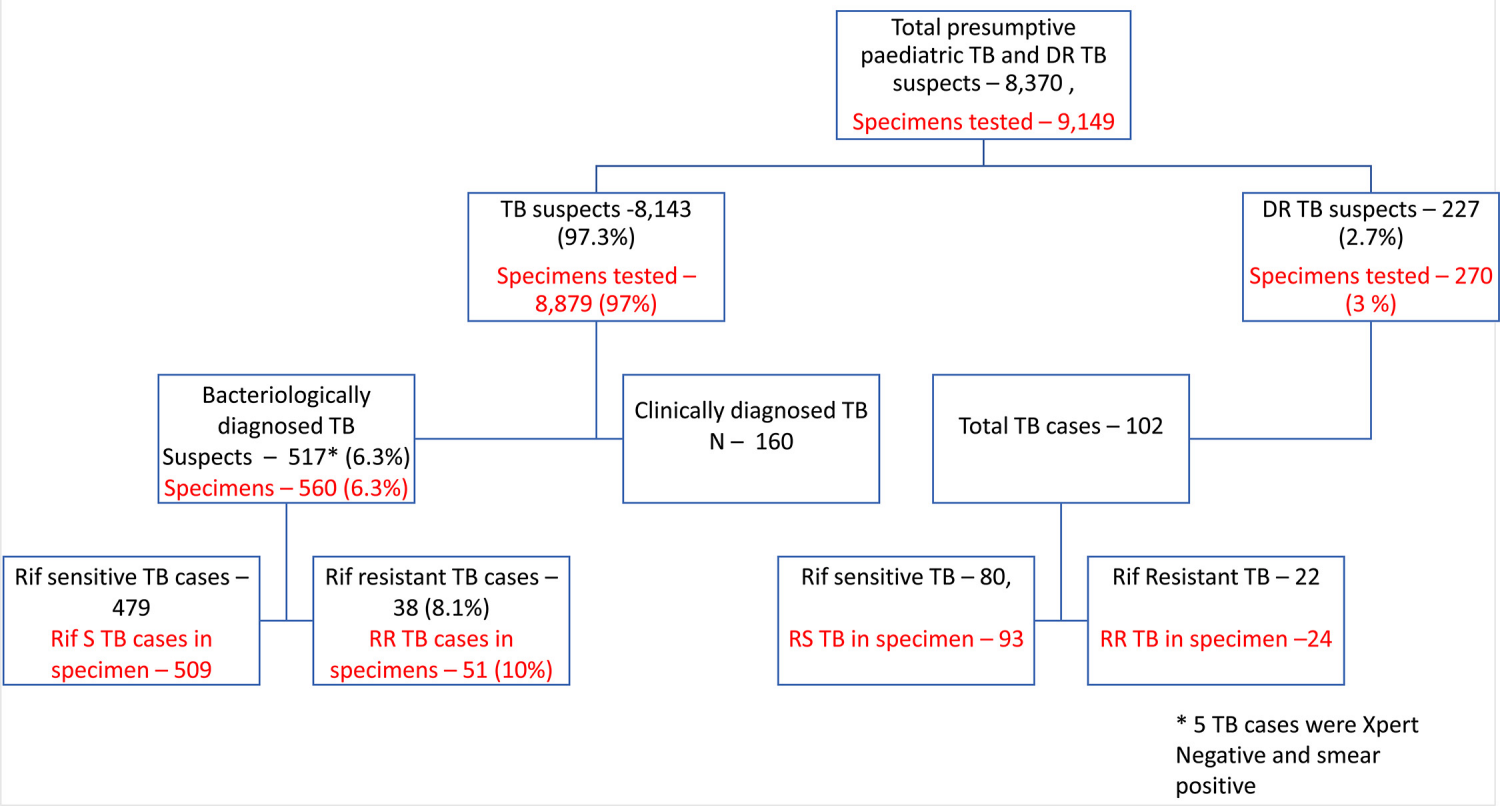
Flow chart of presumptive TB and DR-TB case enrolment and TB detection.

Overall 8,370 paediatric cases were tested in this period, of which, 8,143 (97.3%) were presumptive TB cases and 227 (2.7%) were presumptive DR-TB cases (Fig 2). Of the presumptive TB cases, 4,454 (54.7%) were male and 3,688 (45.3%) were female; 2,355 (28.9%) were in the age-group of 0–4 year, 2,794 (34.3%) in 5–9 years age group and 2,994 (36.8%) in the age group of 10–14 years. Of these, only 201 (2.5%) had past history of anti TB treatment (Table 1).

**Table 1:** Demographic profile of presumptive TB and DR-TB cases.

	Presumptive TB cases	%	Presumptive DR-TB cases	%	Total
Total	8143	97.3%	227	2.7%	**8370**
Gender					
** Female**	3688	45.3%	116	51.1%	3804
** Male**	4454	54.7%	111	48.9%	4565
** TG**	1	0.0%	0	0.0%	1
Age group (yrs)					0
** Below 5**	2355	28.9%	30	13.2%	2385
** 5 to 9**	2794	34.3%	64	28.2%	2858
** 10 to 14**	2994	36.8%	133	58.6%	3127
Past History of anti TB treatment					0
** NA**	749	9.2%	1	0.4%	750
** NO**	7193	88.3%	17	7.5%	7210
** YES**	201	2.5%	209	92.1%	410
Smear Microscopy					0
** NA**	424	5.2%	17	7.5%	441
** Negative**	7553	92.8%	163	71.8%	7716
** Positive**	166	2.0%	47	20.7%	213
No TB	7626	93.7%	125	55.1%	7751
Bacteriologically confirmed TB	517	6.3%	102	44.9%	619
** Xpert-positive; smear-negative**	329	63.6%	48	47.1%	377
** Xpert-positive; smear-NA**	22	4.3%	7	6.9%	29
** Xpert-positive; smear-positive**	161	31.1%	47	46.1%	208
** Xpert-negative; smear-positive**	5	1.0%	0		5
** Xpert-Indeterminate; smear-positive**	0		0		0
** Xpert-NA; smear-positive**	0		0		0

**Fig 3:**
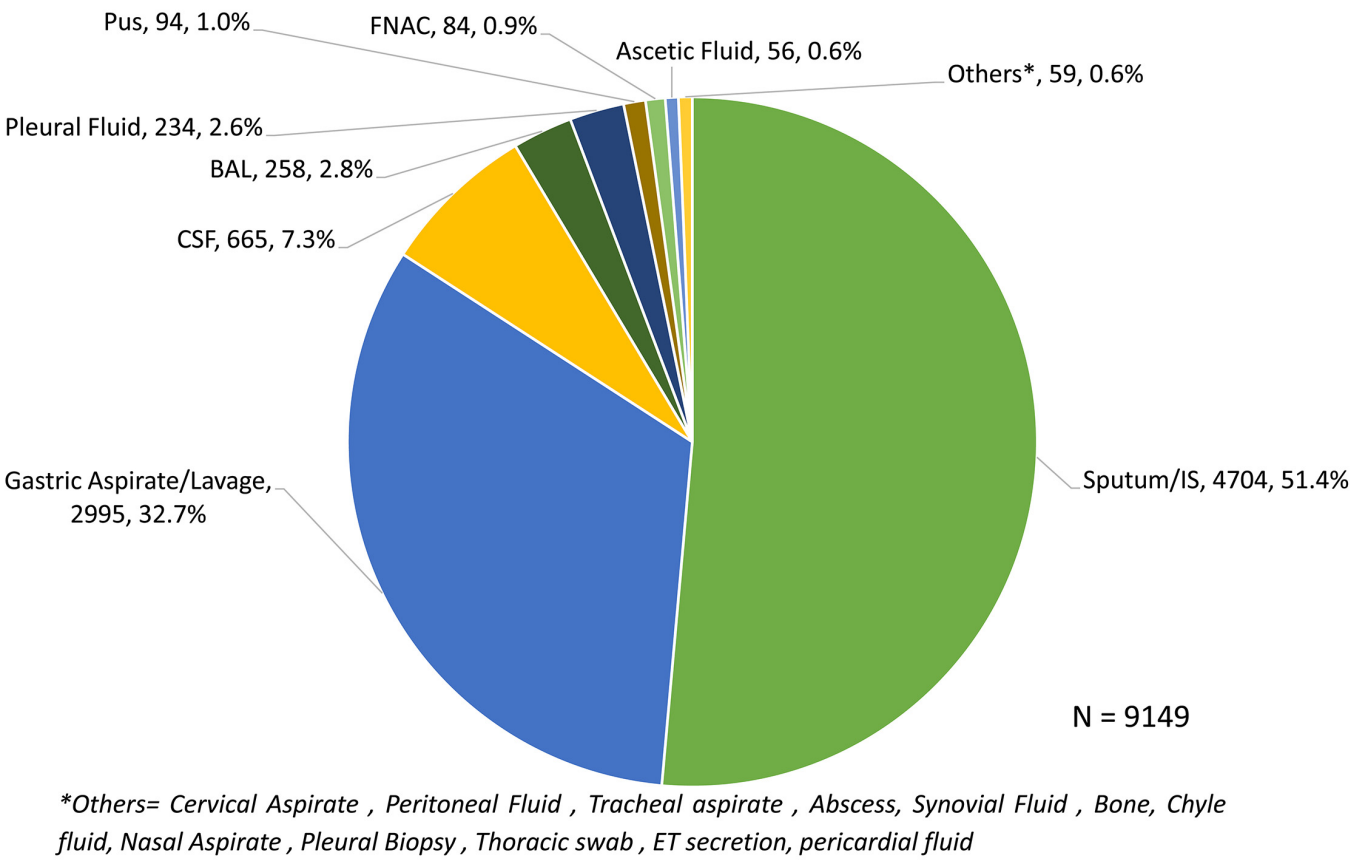
Various types of specimens tested on Xpert MTB/RIF.

A total of 9,149 specimens from 8,370 children were tested and details of various specimen tested on Xpert are listed in Fig 3. Overall 4,445 (48.6%) of the 9,149 specimens tested were non-sputum specimens.

### Feasibility Assessment

Of 9,149 Xpert tests conducted, 8,774 (95.9%, CI 95.4–96.2) valid test result were obtained on a single test. Of the 375 (4.2%, CI 3.8–4.7) initial test failures, repeat testing was done on 332 (88.5%, CI 84.9–91.3) by retesting same or second available specimen. Of these, valid test results on retesting were obtained on 309 (93.0%, CI 89.8–95.3) specimens. Overall, 9,083 (99.2%, CI 99.0–99.4) yielded valid results with 66 (0.7%, CI 0.5–0.9) were test failures. Of the 66 test failures, 43 were not retested due to various programmatic and/or study limitations (Table B in S1 File)

Of the 375 test failures under the pilot, 135 (36%, CI 31.3–40.9) tests failed on account of PCR inhibition giving an invalid test result. Overall invalidity rate observed among different types of specimens tested under the pilot varied from 0% to 2% (Table A in S1 File). Of the total test failure on account of PCR inhibition, majority was observed in sputum (69/135; 51.1%) and gastric aspirate/lavage (53/135; 39.3%). Of these total 135 test failures, 102 (75.6%) were re-tested and 96 (94.1%) were resolved giving a valid result, by retesting a second specimen.

#### Specimen transportation, diagnostic and reporting turnaround time

Specimen of most of the cases, (8,050 (96.2%)), were received on the (same) day of collection while 213 (2.5%) were received a day later. Remaining 1.3% samples were received subsequently due to public holidays. Likewise, test results were made available for 6,718 (80.3%) on the (same) day of sample collection and another 6.6% a day later. Cumulatively, 99.3% (8,314) patients had received their results within a week. Overall, 7,002 (83.7%) of the results were sent to respective providers on the same day of testing and a cumulative total of 7,950 (95.0%) of the results were reported within one day of testing. (Figure B in S1 File)

### Effect of upfront Xpert MTB/RIF on TB case detection

Of the 8,143 presumptive TB cases enrolled, 517 (6.3%, CI 5.8–6.9) were bacteriologically confirmed as TB cases. Of these 517 TB cases, 161 (31.1%) were positive by both Xpert and smear microscopy, while 329 (63.6%) were Xpert TB positive and smear negative; 22 (4.3%) were Xpert positive and smear not done. Additionally, 5 were smear positive and Xpert negative. (Table 1)

TB detection on Xpert was compared against smear microscopy among various types of specimens. Of the total 8,143 presumptive TB cases, smear microscopy was not done for 424 cases. For the remaining 7,719 (94.8%) presumptive TB cases for which both smear and Xpert tests was performed, total of 8,396 different specimens were tested. The additional gain in detection rate on different types of specimen tested under the project on Xpert as compared to microscopy is described in Table 2. Overall, more than 2 fold higher TB case detection was observed on Xpert as compared with smear microscopy irrespective of type of specimen. The increase in TB detection rate was highest while testing CSF, fine needle aspiration cytopathology samples, BAL and Pus specimen with Xpert as compared to smear microscopy (Table 2) Of the, 3,996 various non-sputum specimen tested under the project the overall positivity on Xpert was 299 (7.4%, CI 6.7–8.3), whereas positivity on microscopy was 64 (1.6%, CI 1.2–2.0). It was observed that none of the 52 ascetic fluid specimen tested had a positive test result either on Xpert or smear microscopy. Also, there was no significant gain in TB detection (OR 1.2 (95% CI 0.3–4.0) on Xpert over smear microscopy in case of pleural fluid.

**Table 2:** Additional gain on Xpert MTB/RIF over smear microscopy-specimen wise analysis.

Type of Specimen	Total presumptive TB cases	Cases with simultaneous smear and Xpert MTB/RIF done	Specimen tested	Xpert MTB/RIF Positive	%	Smear Positive	%	Additional gain (Fold)	OR (95% CI)
**Sputum/IS**	4349	4156	4397	237	5.4%	146	3.3%	1.6	1.6 (1.3–2.0)
**Gastric Aspirate/Lavage**	2648	2541	2806	167	6.0%	41	1.5%	4.1	4.2 (3.0–6.0)
**CSF**	551	475	564	40	7.1%	0	0.0%		
**Pleural Fluid**	195	183	186	6	3.2%	5	2.7%	1.2	1.2 (0.3–4.0)
**BAL**	186	180	221	34	15.4%	5	2.3%	6.8	7.8 (3.0–20.4)
**Pus**	73	58	67	24	35.8%	5	7.5%	4.8	6.9 (2.4–19.5)
**FNAC**	54	49	59	21	35.6%	4	6.8%	5.3	7.5 (2.4–23.9)
**Ascetic Fluid**	44	42	52	0	0.0%	0	0.0%	0.0	
**Tissue**	8	7	8	1	12.5%	1	12.5%	1.0	
**Urine**	8	5	6	0	0.0%	0	0.0%	0.0	
**Others** *	27	23	27	6	10.5%	3	5.3%	2.0	2.1 (0.5–8.9)
Total	8143	7719	8396	536	6.4%	210	2.5%	2.6	2.6 2.2–3.1)

*Others = Cervical Aspirate, *Peritoneal Fluid*, *Tracheal aspirate*, *Abscess*, *Synovial Fluid*, *Bone*, *Chyle fluid*, *Nasal Aspirate*, *Pleural Biopsy*, *Thoracic swab*, *ET secretion*, *pericardial fluid*.

### Effect of upfront Xpert MTB/RIF on rifampicin-resistant TB case detection

Of the enrolled 8,143 presumptive paediatric TB cases, total 512 were found positive for TB on Xpert. Of these 38/512 (7.4%, CI 5.4–10.0) were resistant to rifampicin. Additionally, of the 227 presumptive DR-TB cases tested under the pilot, 102 were positive for TB on Xpert, of which 22/102 (21.5%, CI 14.7–30.0) were resistant to rifampicin. Thus, 60 rifampicin resistant TB cases were detected among all patients tested across four sites under this pilot.

Higher proportion of rifampicin resistance was observed in children less than 5 years as compared with children in age group 10–14 years [(12.9% vs 9.1%; OR 1.4 (CI 0.7–2.8)]; more cases detected in males as compared to females [(12.0% vs 8.5%; OR 1.4 (CI 0.8–2.5)] and also in children with past history of TB treatment [(15.6% vs 8.7%; OR 1.9 (CI 1.0–3.4)] (Table 3), though none of these differences were statistically significant.

**Table 3:** Effect of upfront Xpert MTB/RIF on rifampicin-resistant TB case detection.

	Presumptive TB cases	Xpert Positive	Rif Resistant TB		Presumptive DR-TB cases	Xpert Positive	Rif Resistant TB		Total Xpert Positive	Total Rif Resistant TB		OR
			N	%			N	%		N	%	95% CI
**Total**	8143	512	38	7.4%	227	102	22	21.6%	614	60	9.8%	** **
**Age group (yrs)**				** **							** **	
** **Below 5	2355	108	12	11.1%	30	8	3	37.5%	116	15	12.9%	1.4 (0.7–2.8)
** **05–09	2794	122	9	7.4%	64	26	4	15.4%	148	13	8.8%	0.9 (0.4–1.8)
** **10–14	2994	282	17	6.0%	133	68	15	22.1%	350	32	9.1%	1
**Gender**												
** **Female	3688	327	20	6.1%	116	62	13	21.0%	389	33	8.5%	1
** **Male	4454	185	18	9.7%	111	40	9	22.5%	225	27	12.0%	1.4 (0.8–2.5)
** **TG	1	0	0	0.0%	0	0	0		0	0		
**Past history of anti TB**												
** **Unknown	749	27	0	0.0%	1	1	0	0.0%	28	0	0.0%	
** **No	7193	451	37	8.2%	17	7	3	42.9%	458	40	8.7%	1
** **Yes	201	34	1	2.9%	209	94	19	20.2%	128	20	15.6%	1.9 (1.0–3.4)
**Smear Microscopy**												
** **NA	424	22	2	9.1%	17	7	0	0.0%	29	2	6.9%	
** **NEG	7553	329	24	7.3%	163	48	7	14.6%	377	31	8.2%	1
** **POS	166	161	12	7.5%	47	47	15	31.9%	208	27	13.0%	1.6 (0.9–2.8)
**History of contact with TB patient**												
** **Yes			22				17			39		
** **No			8				2			10		
** **Unknown			8				3			11		

Information on history of contact with a known TB/DR-TB patient was collected from all the rifampicin resistant cases. History of contact with a TB / DR-TB case could not be verified for 11/60 (18.3%) patients. In the remaining DR-TB cases, it was observed that, 39/49 (79.6%) had history of contact either with a known TB or DR-TB case. (Table 3) Of these, 39 rifampicin resistant TB cases with positive history of contact, 14 (35.9%) had no prior history of anti TB treatment.

Further analysis was done to assess rifampicin resistant detection rates among various specimens tested under the project. Of the total 9,149 specimens tested, total 677 were found to be positive for TB on Xpert, of which 75 (11.0%, CI 8.9–13.6) were rifampicin resistant. Positivity of rifampicin resistant detection observed in FNAC 7/37 (18.9%, CI 9.4–34.2), CSF 8/46 (17.3%, CI 9.0–30.7), gastric aspirate 25/194 (12.8%, CI 8.8–18.3), BAL 5/41(12.2%, CI 5.3–25.5), pleural fluid 1/9 (11.1%, CI 1.9–43.5), pus 4/39 (10.2%, CI 4.0–23.5) and sputum specimen 25/298 (8.3%, CI 5.7–12.0). (Table C in S1 File)

Of the 60 rifampicin resistant cases diagnosed under the pilot, confirmatory DST could be conducted for 57 cases. Additional or remnant samples from 3 cases were not available for testing (2 cases had died and 1 had defaulted). Of these 57 specimens, valid results were obtained on 40 specimens, of which 36 (90%, CI 76.9–96.4) were found to be rifampicin resistant on LPA or liquid culture DST while 4 (10%, CI 3.9–23) were found to be sensitive to rifampicin.

### Treatment details on confirmed cases

Of the 517 bacteriologically confirmed TB case detected under the study, 38 were resistant to rifampicin. Additional 22 rifampicin resistant cases were detected among 227 presumptive DR-TB cases. Of the total 60 rifampicin resistant TB cases, 51 (85%) patients were initiated on second line treatment, of which 41 (80.4%) were initiated on treatment within 15 days of diagnosis after completing the pre-treatment evaluation as per RNTCP guidelines. Of the total 479 rifampicin sensitive TB patients, 392 (81.8%) patients were initiated on treatment while, 38 (7.9%) patients were initial loss to follow-ups and 11 (2.3%) died. Treatment information for remaining 38 (7.9%) cases was not available as most of them were referrals from private sector and could not be traced. Since patients enrolled under the pilot were from various sectors referred by different providers, diligent follow up on bacteriologically negative TB cases getting diagnosed later on clinical criteria was not feasible hence this aspect has been excluded from current manuscript.

## Discussion

For the first time upfront Xpert testing was offered to all presumptive paediatric TB cases through centralised high through-put Xpert labs in defined geographic areas in India. Participating providers were linked through rapid specimen transportation linkages and rapid result reporting mechanisms. Another novel aspect of this pilot was that, for the first time Xpert testing was extended to non-sputum specimen under routine programmatic conditions in India, in line with the recent WHO recommendations [16]. This led to overall improvement in bacteriologically confirmed paediatric TB cases, as well as detection of significant numbers of rifampicin resistant TB cases in children. All the TB cases diagnosed under the project were notified under RNTCP irrespective of type of referring provider.

While upfront Xpert testing has been recommended by WHO for paediatric presumptive TB cases, implementation of such an intervention is challenging due to high cost of Xpert equipment limiting the test availability across a large number of health facilities; The current pilot project demonstrated the feasibility of rapidly rolling out upfront Xpert testing through a single laboratory exclusively for paediatric population in urban areas by enrolling more than 8,100 paediatric presumptive TB cases within eight months of implementation. Wide geographical coverage of the pilot achieved through rapid specimen transportation linkages with participating health facilities resulting with most specimens being transported on the same day of collection and provided results on the day of specimen collection. This implementation design piloted here provides a feasible methodology of providing upfront access to Xpert, considering that high cost of equipment, limits this test’s applicability for point of care usage covering large geographic areas. Considering that an unexpectedly high proportion of TB cases were found to be resistant to rifampicin, these findings are quite significant in view of much higher turnaround time on other available diagnostic tests for the diagnosis of drug resistance [10].

For wider geographic coverage a large number of sensitization workshops and advocacy meetings were organised with various health providers. These activities led to enhanced referrals from various providers and overall increase in presumptive TB case enrolment under the project. Though objective before and after comparative data could not be documented for the same, with the availability of free services, door to door pick up of specimens from linked heath providers and reporting of test results in a time bound manner, the pilot resulted in large number of providers utilizing these free Xpert testing services. All diagnosed cases were given an option of getting free treatment services and case management under RNTCP.

Under this pilot, Xpert testing for first time was extended to various types of non-sputum and non-respiratory specimens to assess the performance of this assay under uncontrolled field conditions. Xpert performance on both sputum and non-sputum was found to be highly satisfactory, with overall 99.3% cases getting valid results. These findings are similar to other studies conducted on Xpert assay on sputum [21–24] and non-sputum specimens [25]. However, proportion of interpretable results on Xpert obtained under the current study was observed to be higher than previous reports from India and Germany [10, 26]. The key factor contributing to the high proportion of interpretable results was rapid retesting thereby resolving test failures on the same specimen. This was similar to earlier reports from India [21].

Polymerase chain reaction inhibition leading to invalid test results is a major concern while testing specimens on various types of molecular assays, especially non-sputum specimen [27–29]. The invalidity rate observed in our study on account of PCR inhibition was lower than reported in another study on Xpert assay from India [10] and on other PCR based assays [30]. Invalid or false negative results in various PCR based tests are mostly due to the presence of inhibitors, sub-optimal assay conditions or omission of key steps [31]. However, this issue was seen to be of lesser concern on Xpert which can be attributed to the fact that this test automated and self-contained test that offers minimal hands-on manual manipulation of specimen which may be a key factor in leading to low PCR inhibition rates.

The current pilot assessed the diagnostic yield of Xpert, a test with already documented high specificity, in comparison to smear under routine programmatic conditions. The current study design limited the possibility of additional, parallel testing of the collected specimen on liquid culture, which might have provided useful performance information such as sensitivity and specificity of the test. However, the same was beyond the scope of the current pilot. Xpert detected more than twice TB cases over smear microscopy, these findings suggest better sensitivity of Xpert for TB diagnosis than smear microscopy in children. Our findings are in-line with the findings from recent meta-analysis by Anne Detjen et al [32]. Xpert performance in TB detection was excellent among various specimens tested as majority of specimen has yielded valid results. However, higher positivity was observed in specimens such as gastric aspirate/gastric lavage, CSF, FNAC, Pus and BAL specimens. These findings are similar to findings from other study conducted in India [10]. Our observation is similar to the findings from other similar studies from different countries [26, 33–38]. The positivity rate on ascetic fluid and pleural fluid observed in our pilot was very low, in-line with guidance issued by WHO [16] and observations in different studies [39–41] which suggests limited utility of testing these specimen on Xpert over and above smear microscopy.

By offering upfront Xpert to all presumptive TB and DR-TB cases, substantial numbers of rifampicin resistant TB patients were diagnosed under the study. Though limited data on levels of rifampicin resistance in paediatric population is available, our study findings are broadly similar to the findings from earlier studies conducted in India in the same age group [36, 42–43]. The levels of rifampicin resistance were high in all three age groups, i.e. 0–4, 5–9 & 10–14. Further, around half of the diagnosed rifampicin resistance cases were smear negative. Other WHO endorsed rapid tests for the diagnosis of rifampicin resistance have limited diagnostic utility on smear negative specimens [31, 20]. Though the current pilot was not designed to draw a representative estimate of rifampicin resistance levels in the diagnosed paediatric TB cases, the findings underscore the importance of considering upfront Xpert testing for presumptive TB cases in this highly vulnerable population.

The data from the current pilot shows that rifampicin resistance in paediatric TB cases correlated better with a positive history of contact, rather than a past history of TB treatment. We did not observe any correlation between the detection of rifampicin resistant and BCG vaccination status. While most of the rifampicin resistant TB cases had a positive history of contact with TB cases, a large proportion of them had no prior history of TB treatment. This finding suggests that history of contact with a TB patient can be considered as a more appropriate risk factor for rifampicin resistance in paediatric population, rather than just focusing on past history of TB treatment.

While cost implications are always important in implementation, the present study focuses solely on assessing the feasibility and impact of upfront Xpert testing on the pediatric TB & DR-TB detection rates. Results of this approach can contribute to the basis for assessment of cost-effectiveness. As a logical next step costing of the project interventions is being undertaken by the project team findings of which will be published separately.

## Conclusion

Xpert with advantages of quick turnaround testing-time, high proportion of interpretable results and feasibility of rapid rollout, provides a promising solution to the TB diagnostic challenges in children, while simultaneously detecting rifampicin resistant TB. The pilot demonstrated the feasibility of extending Xpert testing to non-sputum specimens with a very high proportion of interpretable results (similar to sputum specimen) and the feasibility of linking a large number of providers with a single Xpert lab, efficiently. With more than a two fold increase in TB case detection over smear microscopy and detection of significant numbers of rifampicin resistant TB cases, the study demonstrates the utility of offering upfront Xpert testing to paediatric presumptive TB and DR-TB patients under programmatic conditions.

## Supporting Information

S1 File Table A. Xpert MTB/RIF test performance. Table B. Test failure rate on different type of specimens. Table C. Specimen wise Rifampicin resistant Positivity. Figure A. Rapid scale up across four sites. Figure B. Transportation, Diagnosis and Reporting Turnaround Time. (DOCX)
