# Supplementary material for: Piloting Upfront Xpert MTB/RIF Testing on Various Specimens under Programmatic Conditions for Diagnosis of TB & DR-TB in Paediatric Population
Source: PLoS One. 2015 Oct 15;10(10):e0140375. doi: 10.1371/journal.pone.0140375 (PMC4607299; doi:10.1371/journal.pone.0140375)
Supplement: S1 File — (DOCX) [file pone.0140375.s001.docx]

**Supporting Information**

**Table A:** **Xpert MTB/RIF test performance**

| **Type of Specimen** | **Grand Total** | **Error** | **Retested** | **Resolved** | **Invalid** | **Retested** | **Resolved** | **No Result** | **Retested** | **Resolved** | **Total Test Failure** | **Total Retested** | **Total Resolved** | **% Error** | **% Invalid** | **%No result** |
| --- | --- | --- | --- | --- | --- | --- | --- | --- | --- | --- | --- | --- | --- | --- | --- | --- |
| Sputum/IS | 4704 | 105 | 101 | 97 | 71 | 56 | 51 | 19 | 19 | 19 | 195 | 176 | 167 | 2.2% | 1.5% | 0.4% |
| Gastric Aspirate/Lavage | 2995 | 83 | 79 | 67 | 60 | 45 | 44 | 2 | 2 | 2 | 145 | 126 | 113 | 2.8% | 2.0% | 0.1% |
| CSF | 665 | 12 | 12 | 12 | 2 | 1 | 1 | 2 | 1 | 1 | 16 | 14 | 14 | 1.8% | 0.3% | 0.3% |
| BAL | 258 | 7 | 6 | 5 | 1 | 0 | 0 | 2 | 2 | 2 | 10 | 8 | 7 | 2.7% | 0.4% | 0.8% |
| Pleural Fluid | 234 | 5 | 5 | 5 | 0 | 0 | 0 | 0 | 0 | 0 | 5 | 5 | 5 | 2.1% | 0.0% | 0.0% |
| Pus | 94 | 1 | 1 | 1 | 1 | 0 | 0 | 0 | 0 | 0 | 2 | 1 | 1 | 1.1% | 1.1% | 0.0% |
| FNAC | 84 | 1 | 1 | 1 | 0 | 0 | 0 | 0 | 0 | 0 | 1 | 1 | 1 | 1.2% | 0.0% | 0.0% |
| Ascetic Fluid | 56 | 0 | 0 | 0 | 0 | 0 | 0 | 1 | 1 | 1 | 1 | 1 | 1 | 0.0% | 0.0% | 1.8% |
| Tissue | 10 | 0 | 0 | 0 | 0 | 0 | 0 | 0 | 0 | 0 | 0 | 0 | 0 | 0.0% | 0.0% | 0.0% |
| Urine | 9 | 0 | 0 | 0 | 0 | 0 | 0 | 0 | 0 | 0 | 0 | 0 | 0 | 0.0% | 0.0% | 0.0% |
| Pericardial Fluid | 8 | 0 | 0 | 0 | 0 | 0 | 0 | 0 | 0 | 0 | 0 | 0 | 0 | 0.0% | 0.0% | 0.0% |
| Synovial Fluid | 6 | 0 | 0 | 0 | 0 | 0 | 0 | 0 | 0 | 0 | 0 | 0 | 0 | 0.0% | 0.0% | 0.0% |
| Cervical Aspirate | 5 | 0 | 0 | 0 | 0 | 0 | 0 | 0 | 0 | 0 | 0 | 0 | 0 | 0.0% | 0.0% | 0.0% |
| Peritoneal Fluid | 5 | 0 | 0 | 0 | 0 | 0 | 0 | 0 | 0 | 0 | 0 | 0 | 0 | 0.0% | 0.0% | 0.0% |
| Abscess | 4 | 0 | 0 | 0 | 0 | 0 | 0 | 0 | 0 | 0 | 0 | 0 | 0 | 0.0% | 0.0% | 0.0% |
| Tracheal Aspirate | 4 | 0 | 0 | 0 | 0 | 0 | 0 | 0 | 0 | 0 | 0 | 0 | 0 | 0.0% | 0.0% | 0.0% |
| Nasal Aspirate | 2 | 0 | 0 | 0 | 0 | 0 | 0 | 0 | 0 | 0 | 0 | 0 | 0 | 0.0% | 0.0% | 0.0% |
| Pleural Biopsy | 2 | 0 | 0 | 0 | 0 | 0 | 0 | 0 | 0 | 0 | 0 | 0 | 0 | 0.0% | 0.0% | 0.0% |
| Bone | 1 | 0 | 0 | 0 | 0 | 0 | 0 | 0 | 0 | 0 | 0 | 0 | 0 | 0.0% | 0.0% | 0.0% |
| Chyle Fluid | 1 | 0 | 0 | 0 | 0 | 0 | 0 | 0 | 0 | 0 | 0 | 0 | 0 | 0.0% | 0.0% | 0.0% |
| ET Secretion | 1 | 0 | 0 | 0 | 0 | 0 | 0 | 0 | 0 | 0 | 0 | 0 | 0 | 0.0% | 0.0% | 0.0% |
| Thoracic secretion | 1 | 0 | 0 | 0 | 0 | 0 | 0 | 0 | 0 | 0 | 0 | 0 | 0 | 0.0% | 0.0% | 0.0% |
| **Grand Total** | 9149 | 214 | 205 | 188 | 135 | 102 | 96 | 26 | 25 | 25 | 375 | 332 | 309 | 2.3% | 1.5% | 0.3% |

**Table B: Test failure rate on different type of specimens**

| Type of Specimen | Total specimen tested | Valid results | % valid results | Initial test Failure | % Test failures | Retested | Resolved | Final valid | % | Final test failure | % |
| --- | --- | --- | --- | --- | --- | --- | --- | --- | --- | --- | --- |
| Sputum/IS | 4704 | 4509 | 95.9% | 195 | 4.1% | 176 | 167 | 4676 | 99.4% | 28 | 0.6% |
| Gastric Aspirate/Lavage | 2995 | 2850 | 95.2% | 145 | 4.8% | 126 | 113 | 2963 | 98.9% | 32 | 1.1% |
| CSF | 665 | 649 | 97.6% | 16 | 2.4% | 14 | 14 | 663 | 99.7% | 2 | 0.3% |
| BAL | 258 | 248 | 96.1% | 10 | 3.9% | 8 | 7 | 255 | 98.8% | 3 | 1.2% |
| Pleural Fluid | 234 | 229 | 97.9% | 5 | 2.1% | 5 | 5 | 234 | 100.0% | 0 | 0.0% |
| Pus | 94 | 92 | 97.9% | 2 | 2.1% | 1 | 1 | 93 | 98.9% | 1 | 1.1% |
| FNAC | 84 | 83 | 98.8% | 1 | 1.2% | 1 | 1 | 84 | 100.0% | 0 | 0.0% |
| Ascetic Fluid | 56 | 55 | 98.2% | 1 | 1.8% | 1 | 1 | 56 | 100.0% | 0 | 0.0% |
| Tissue | 10 | 10 | 100.0% | 0 | 0.0% | 0 | 0 | 10 | 100.0% | 0 | 0.0% |
| Urine | 9 | 9 | 100.0% | 0 | 0.0% | 0 | 0 | 9 | 100.0% | 0 | 0.0% |
| Pericardial Fluid | 8 | 8 | 100.0% | 0 | 0.0% | 0 | 0 | 8 | 100.0% | 0 | 0.0% |
| Synovial Fluid | 6 | 6 | 100.0% | 0 | 0.0% | 0 | 0 | 6 | 100.0% | 0 | 0.0% |
| Cervical Aspirate | 5 | 5 | 100.0% | 0 | 0.0% | 0 | 0 | 5 | 100.0% | 0 | 0.0% |
| Peritoneal Fluid | 5 | 5 | 100.0% | 0 | 0.0% | 0 | 0 | 5 | 100.0% | 0 | 0.0% |
| Abscess | 4 | 4 | 100.0% | 0 | 0.0% | 0 | 0 | 4 | 100.0% | 0 | 0.0% |
| Tracheal Aspirate | 4 | 4 | 100.0% | 0 | 0.0% | 0 | 0 | 4 | 100.0% | 0 | 0.0% |
| Nasal Aspirate | 2 | 2 | 100.0% | 0 | 0.0% | 0 | 0 | 2 | 100.0% | 0 | 0.0% |
| Pleural Biopsy | 2 | 2 | 100.0% | 0 | 0.0% | 0 | 0 | 2 | 100.0% | 0 | 0.0% |
| Bone | 1 | 1 | 100.0% | 0 | 0.0% | 0 | 0 | 1 | 100.0% | 0 | 0.0% |
| Chyle Fluid | 1 | 1 | 100.0% | 0 | 0.0% | 0 | 0 | 1 | 100.0% | 0 | 0.0% |
| ET Secretion | 1 | 1 | 100.0% | 0 | 0.0% | 0 | 0 | 1 | 100.0% | 0 | 0.0% |
| Thoracic secretion | 1 | 1 | 100.0% | 0 | 0.0% | 0 | 0 | 1 | 100.0% | 0 | 0.0% |
| Grand Total | **9149** | **8774** | **95.9%** | **375** | **4.1%** | **332** | **309** | **9083** | **99.3%** | **66** | **0.7%** |

**Table C: Specimen wise Rifampicin resistant Positivity**

| Type of Specimen | Total specimens tested | Total TB case diagnosed on Xpert MTB/RIF | | Total Rifampicin resistant cases | |
| --- | --- | --- | --- | --- | --- |
|  |  | **N** | **%** | **N** | **%** |
| Sputum/IS | 4704 | 298 | 6.3% | 25 | 8.4% |
| Gastric Aspirate/Lavage | 2995 | 194 | 6.5% | 25 | 12.9% |
| CSF | 665 | 46 | 6.9% | 8 | 17.4% |
| BAL | 258 | 41 | 15.9% | 5 | 12.2% |
| Pleural Fluid | 234 | 9 | 3.8% | 1 | 11.1% |
| Pus | 94 | 39 | 41.5% | 4 | 10.3% |
| FNAC | 84 | 37 | 44.0% | 7 | 18.9% |
| Ascetic Fluid | 56 | 0 | 0.0% | 0 | 0.0% |
| Tissue | 10 | 2 | 20.0% | 0 | 0.0% |
| Urine | 9 | 0 | 0.0% | 0 | 0.0% |
| Others* | 40 | 11 | 27.5% | 0 | 0.0% |
| Total | 9149 | 677 | 7.4% | 75 | 11.1% |

**Others= Cervical Aspirate , Peritoneal Fluid , Tracheal aspirate , Abscess, Synovial Fluid , Bone, Chyle fluid, Nasal Aspirate , Pleural Biopsy , Thoracic swab , ET secretion, pericardial fluid*

**Figure A: Rapid scale up across four sites**

**
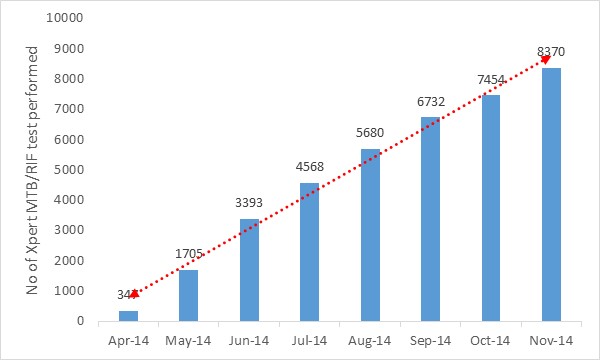
**

**Figure B: Transportation, Diagnosis and Reporting Turnaround Time**

**
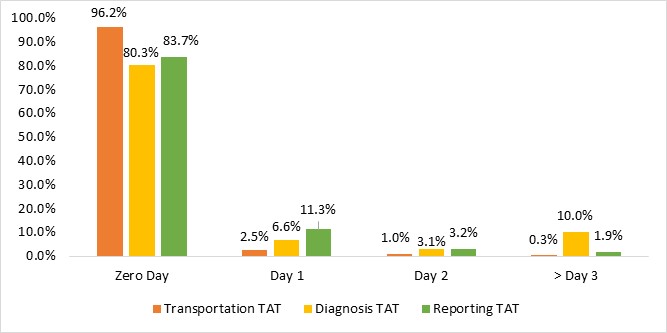
**
